# Blue Whales Respond to Anthropogenic Noise

**DOI:** 10.1371/journal.pone.0032681

**Published:** 2012-02-29

**Authors:** Mariana L. Melcón, Amanda J. Cummins, Sara M. Kerosky, Lauren K. Roche, Sean M. Wiggins, John A. Hildebrand

**Affiliations:** Scripps Institution of Oceanography, University of California San Diego, La Jolla, California, United States of America; University of Saint-Etienne, France

## Abstract

Anthropogenic noise may significantly impact exposed marine mammals. This work studied the vocalization response of endangered blue whales to anthropogenic noise sources in the mid-frequency range using passive acoustic monitoring in the Southern California Bight. Blue whales were less likely to produce calls when mid-frequency active sonar was present. This reduction was more pronounced when the sonar source was closer to the animal, at higher sound levels. The animals were equally likely to stop calling at any time of day, showing no diel pattern in their sensitivity to sonar. Conversely, the likelihood of whales emitting calls increased when ship sounds were nearby. Whales did not show a differential response to ship noise as a function of the time of the day either. These results demonstrate that anthropogenic noise, even at frequencies well above the blue whales' sound production range, has a strong probability of eliciting changes in vocal behavior. The long-term implications of disruption in call production to blue whale foraging and other behaviors are currently not well understood.

## Introduction

The use of sound for communication and acquisition of information about the environment has evolved across the years and constitutes an important aspect of baleen whale behavior [1]. Given the increasing level of anthropogenic noise in the ocean [2], there has been concern that high-intensity anthropogenic noise may impact communication and other behaviors involving whale sound production [3]–[7], especially when the frequencies of the animals' calls and the noise overlap. It may be intuitive to think of a potential impact of noise in the same frequency band that animals use, for example, through masking. However, the impact of non-overlapping noise has received less attention. To our knowledge, there are no published studies addressing the impact of mid-frequency anthropogenic noise on baleen whales, where the frequency ranges produced by the sound source and the animals do not overlap.

Recently, McKenna and colleagues [8] found that blue whale song was disrupted in the presence of ships. Additionally, foraging animals showed a partial Lombard effect [9], which means that the amplitude of the calls increased to keep a high signal to noise ratio. Their study [8], however, was conducted focusing in the low frequencies of the ship noise, which overlap with the whale's vocalizations. Likewise, Miller and colleagues [10] found that playback of low-frequency active (LFA) sonar elicited lengthening of humpback whales' songs.

One population of blue whales (*Balaenoptera musculus*), an endangered species [11], is encountered during the summer in the Southern California Bight. This population typically produces distinct low-frequency (<100 Hz) sounds (D calls) associated with foraging behavior [12]–[13]. Tag data revealed that these calls are produced by both male and female blue whales, but only in a foraging context [13]. These calls are believed to attract other individuals to feeding grounds or maintain cohesion within the foraging group [14]. Given the conservation status of blue whales and the current concern about potential effects of man-made noise on marine mammals, the aim of the present study was to determine whether anthropogenic noise in the mid-frequency range (1–8 kHz) elicited a behavioral response in blue whales and, if so, whether there was a particular time of the day at which animals were more prone to react to those anthropogenic sources. Here we found during the foraging season for two consecutive years (2009 and 2010) that blue whales responded significantly to MFA sonar and ship noise. However, we found no particular time of the day during which animals were more prone to react to either anthropogenic noise source.

## Results

### Is there a behavioral response of blue whales to anthropogenic noise?

To test whether the D call production of blue whales was affected by anthropogenic noise in the mid-frequency band, we used passive acoustic monitoring data recorded with High-frequency Acoustic Recording Packages (HARPs) [15] in the Southern California Bight (Table 1) and analyzed the presence of D calls as well as MFA sonar events. The experimental site was close to a naval training area; therefore, we expected to opportunistically record MFA sonar events both nearby (high received levels) and far away (low received levels) in the MFA sonar frequency range (1–8 kHz). We also analyzed explosions and ship propulsion events occurring in that same frequency band. In the case of the latter sources, however, it is understood that they contain more energy at low frequencies, but that the presence of mid-frequency energy suggests that they are located nearby the acoustic receiver.

**Table 1:** Characteristics of the study site.

	Study site
Latitude	33°22.0′N
Longitude	118°34.0′W
Depth	1300 m
Effort	4643 h
Time with MFA	9%
Time with ship noise	27%
Time with explosions	1%
Time with D calls	48%
P (D calls | MFA)	0.28
P (D calls | ship)	0.43
P (D calls | explosions)	0.33
P (D calls | non-anthro)	0.52

The times represent the percentage of hours containing noise or D calls out of the total amount of hours analyzed.

**Figure 1:**
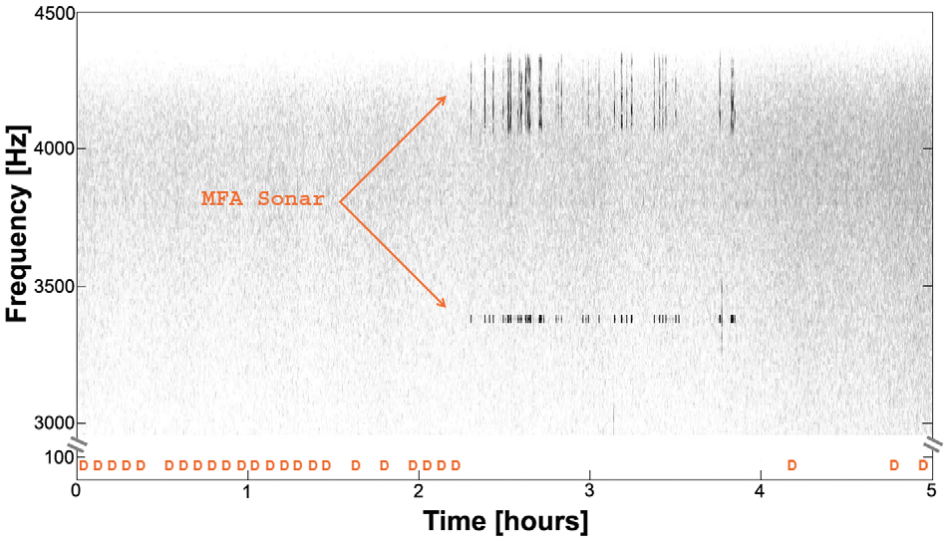
Example of D calls in presence of MFA sonar. Long-term spectral average of 5 hours. Each orange “D” represents presence of D calls in 5-minute bins in the lower frequency band (25–100 Hz). Note the continuous presence of D calls for over 2 hours until the onset of MFA sonar (not a particularly close event, with signals every 10–30 seconds), at which time at which the whales cease production of D calls. After sonar cessation, blue whales start producing D calls again.

We restricted our analysis to the feeding seasons of 2009 and 2010, i.e. to the months of June–August, and calculated the probability of D calls given MFA sonar (P (D calls | MFA)), the probability of D calls given ship noise (P (D calls | ship)), the probability of D calls given explosions (P (D calls | explosions)), and the probability of D calls given ambient (non-anthropogenic) noise (P (D | non-anthropogenic)). Non-anthropogenic noise comes from natural abiotic sources such as wind, rain and earthquakes, and from biotic sources such as dolphin vocalizations. The ratio between the probability of calling in the presence of each anthropogenic noise divided by the P (D | non-anthropogenic) provided us with information on the probability of recording D calls, using the “non-anthropogenic noise” situation as a reference or baseline. Here we found that for MFA sonar, the ratio was 0.54, meaning that the P (D calls| MFA) was almost half of the P (D calls| non-anthropogenic). As an example of this response, we show a time series with D call cessation when MFA sonar was detected (Fig. 1). Similarly, the ratio for the explosions was 0.63, although the sample size is rather small (N = 51). Finally, the ratio for ship noise was 0.83, meaning that P (D calls | ships) was more similar to P (D | non-anthropogenic).

### Whale's response as a function of received level in the mid-frequency band

**Figure 2:**
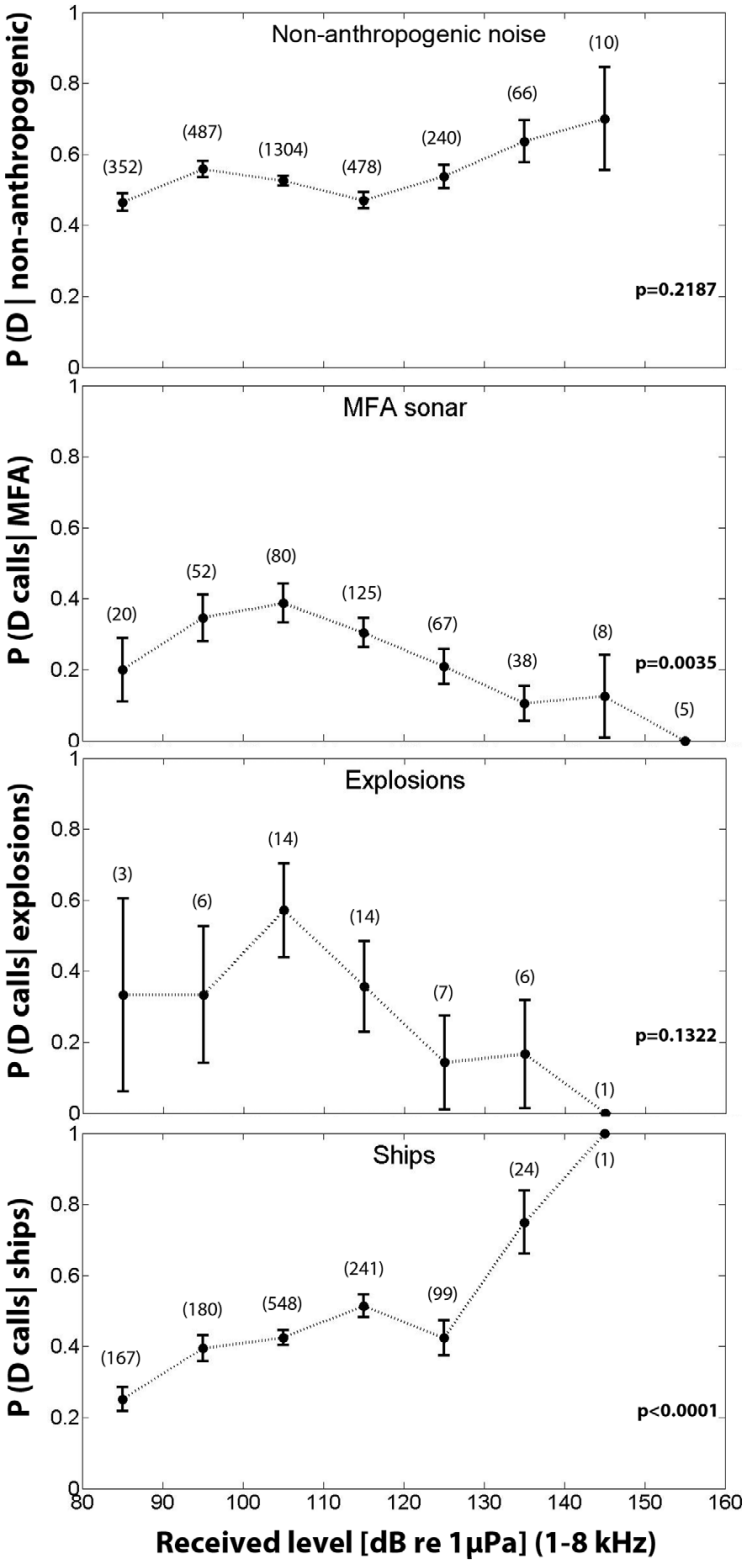
Probability of D calls as a function of SPL. Proportion of hours containing D calls ± s.e. as a function of the maximum sound pressure level (rms) of each hour for non-anthropogenic noise, MFA sonar, explosions and ship noise. P values are given for each condition and parentheses represent the number of hours contributing to each data point. Whereas, the probability of D calls given non-anthropogenic noise and explosions showed no significant dependency on the received level, the probability of MFA sonar decreased with increasing received levels. The probability of D calls given ship noise, on the contrary, increased as a function of the SPL in the mid-frequency range.

To test whether the presence of D calls depended on the intensity level of noise in the mid-frequency range, we calculated the recorded sound pressure level (SPL). To do so, we calculated the root of the mean of the squared pressure (rms), second by second, in the frequency band of MFA sonar (1–8 kHz) throughout the whole blue whale feeding season. We plotted the proportion of hours with D calls as a function of the maximum SPL calculated for each hour. We repeated this procedure for ship noise and explosions. The remaining hours without anthropogenic noise were labelled as “non-anthropogenic noise” and included storms, wind and dolphin vocalizations. Logistic regressions [16] were calculated with a maximum likelihood approach and the corresponding p values are shown in Fig. 2 for each situation.

We found that the probability of D calls given non-anthropogenic noise did not show a significant change as a function of the SPL (p = 0.2187). In contrast, the probability of D calls given MFA sonar decreased significantly with increasing received level (p = 0.0035). Besides, the likelihood of detecting D calls when MFA sonar was present was in general lower than the probability of D calls given non-anthropogenic noise. This speaks for an overall effect of the presence of MFA sonar events in the probability of D calls recorded.

When explosions were analyzed, although the probability of D calls seemed to decrease with increasing SPL (Fig. 2), the statistical analysis showed no significant differences (p = 0.1322). Finally, the probability of D calls given ship noise increased significantly as a function of the received level, showing the opposite effect from MFA sonar and explosions.

### Diel pattern of D calls

**Figure 3:**
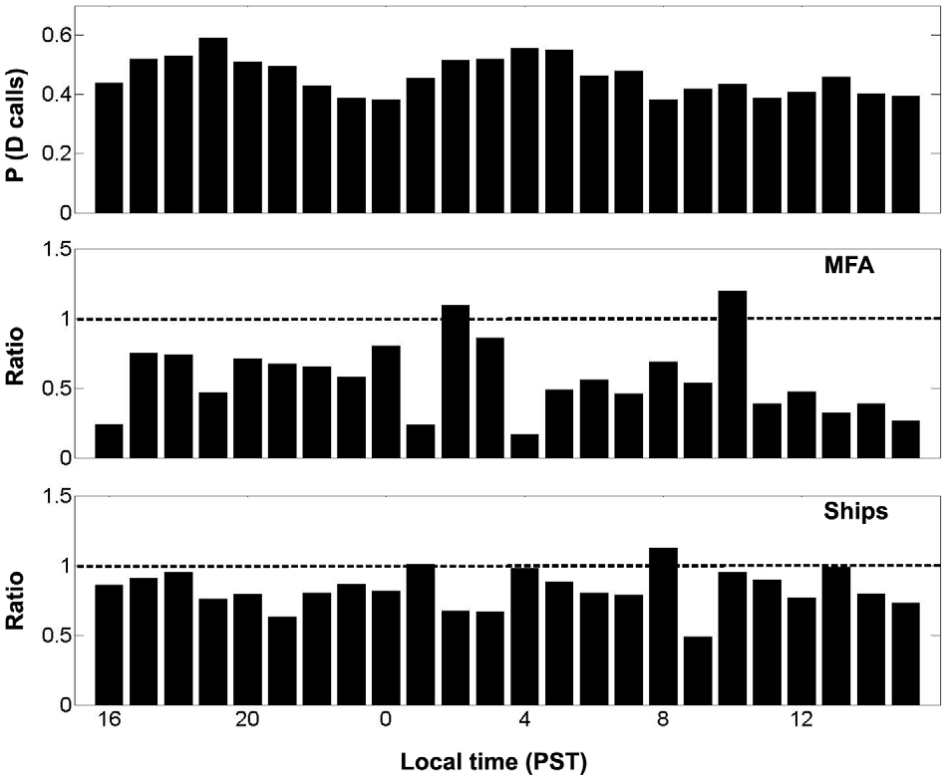
Diel pattern of D calls and sensitivity to MFA sonar. Upper panel represents the probability of D calls as a function of the time of the day for both feeding seasons (2009–2010). Middle and lower panels show, respectively, the ratio of the probability of D calls given MFA sonar and the probability of D calls given ship noise divided by the probability of D calls given non-anthropogenic noise as a function of the time. Values below 1 indicate a lower incidence of D calls given MFA sonar or ship noise; whereas, values above 1 indicate the opposite.

Next, we analyzed the diel pattern of D calls to test whether animals were more prone to react to MFA sonar or ship noise at a particular time of the day. For this, we first plotted the probability of D calls per hour in total (Fig. 3, upper panel) to obtain a baseline of the D call production. Here we found a similar diel pattern to the one reported by Wiggins *et al* [17] and Oleson *et al* [14], where animals produced D calls throughout the day, with two clear peaks of increased calling: one during sunset and the other one shortly before sunrise. Next, we wanted to know whether animals were more sensitive to anthropogenic noise at a particular time of the day. When looking at the ratios of P (D calls | MFA) or P (D calls | ship noise) divided by P (D | non-anthropogenic) per hour (Fig. 3, middle and lower panels) we did not find any clear tendency for the whales to be affected by either anthropogenic noise source at particular times of the day. Note that, since we used the ratios instead of P (D calls) for the middle and lower panels, we have corrected for the relative D call activity at each time of the day.

## Discussion

### MFA sonar

In the present work, we showed that blue whales decreased the proportion of time spent producing D calls to half when MFA sonar was present. Moreover, this cessation of calling depended on the intensity of noise in the mid-frequency range. These results represent a lower boundary for this correlation for two reasons. First, our analysis is based on presence or absence of whales and sonar every hour, which results in a conservative approach. An animal could have produced D calls until the onset of an MFA sonar event and, if both occurred within the same hour, our methodology would consider this situation as an overlap, resulting in a “no response” assignment, although there might have been a reaction (e.g. Fig. 1). Second, even though we do not know the exact locations of the whale and the MFA sonar source, our empirical model (see Fig. S1, upper panel) indicates that the received levels at the recorder are about 20 dB lower than at the whale when the MFA sonar source is beyond 8 km. The distribution of received levels (see Fig. S1, lower panel) suggests that the sonar source was never closer than 6–8 km (i.e. <160 dB re 1 µPa). If we recorded the MFA sonar SPL at the animal, however, the correlation may be strengthened because of the higher levels at the animal, but the behavioral patterns observed should not change. In other words, we may not know the exact SPL at the whale, but the end result seems clear: the closer the MFA source (i.e. the higher the levels at the animal), the lower the probability of recording D calls.

While the anthropogenic noise sources analyzed here are not in the frequency range of most baleen whale calls, a response by blue whales to MFA sonar suggests that they have the ability to perceive these sounds, as suggested by Erbe [6] and Southall et al. [7]. One possibility for the adaptive value of the extended hearing range in blue whales is that it may be advantageous, for instance, to hear their predators, i.e. killer whales, which vocalize in the same frequency range as MFA sonar [18]–[19].

When the sonar source is close to the whale (≤2 km), the SPL at the animal can be substantially higher than recorded at the seafloor HARP. Although there are no empirical data on SPL causing temporary threshold shift (TTS) in baleen whales, Southall et al. [7] estimated its onset at 215 dB. Even if MFA sonar were not causing TTS, we do not know if the suppression of D calls reflects a change in the feeding performance or the abandonment of the foraging behavior.

It is remarkable that relatively low intensity sound levels cause a perturbation such that the probability of D calls decreases (Fig. 2) compared to our reference (non-anthropogenic noise). This suggests that a single MFA sonar source could elicit a response from blue whales over a broad region of the Southern California Bight.

### Explosions and ship noise

Explosions, which are impulsive noise events and contain the majority of their energy content in lower frequencies, showed a similar effect on foraging calls as the MFA sonar relative to the received levels, i.e. a decrease of the probability of D calls with increasing received level in the mid-frequency band. However, we did not find any significant differences, probably due to the low sample size.

Ship noise consists of broadband noise, but with the majority of the energy at low frequencies. Typically, the noise of a ship passing by can last for tens of minutes. This means the probability of detecting D calls when ships sounds are also present should decrease, since the call frequencies overlap with the ship noise and could be easily masked by it. However, to our surprise, the probability of D calls given ship noise increased with increasing SPL. This result suggests one contribution may be the vocal response of the animals to overcome the noise so that they still will be able to communicate with each other (i.e. Lombard effect) which would increase the whale source level to match the increased ship noise. However, since the probability of D calls increases with ship noise received levels, other factors may also be contributing to the increased probability of D calls. For example, one possibility is that the received noise level at the whale is higher than at the HARP for nearby ships, creating a greater apparent vocal response by the whale than the ship noise level increase at the HARP.

This result is in agreement with a previous study [8], in which the authors found blue whales emitting D calls with higher source levels when ships were passing by, showing a Lombard effect [9]. Furthermore, they found a higher proportion of multiple callers when ships were present. Multiple callers during ship noise may be another factor explaining our observed increase in calling in the presence of ships.

### Diel patterns

Since the animals responded homogenously or without a consistent diel pattern to MFA sonar or ship noise (Fig. 3), it suggests no time-dependent sensitivity to the noise. Yet, the consistent diel pattern observed for D calls leads one to infer that the impact of MFA sonar and ship noise may be greater during sunset and shortly before sunset, at least in the studied behavioral context.

### Conclusions

Our data show an acoustical response from blue whales to MFA sonar and ship noise. In particular, there is a disruption of the D call production of these animals with MFA sonar. The implications of such a response are unknown to date, but owing to the low received level, a single source of MFA sonar may be capable of affecting the animals' vocal behavior over a substantial area. Additionally, nearby ships elicit more intense D calling by blue whales. More research is encouraged to understand the effects of anthropogenic noise exposure at the individual and population level.

## Materials and Methods

### Data acquisition

One HARP [15] deployment site was studied in the Southern California Bight (Table 1). The instrument recorded continuously at a sampling rate of 200 kHz for 2–4 months per deployment, for a total of 4 deployments over two consecutive blue whale feeding seasons. After recovery, data were stored as wav files and decimated by a factor of 20 (for mid-frequency) or 100 (for low-frequency) for analysis. Trained analysts manually logged blue whale D calls and MFA sonar events, explosions, and ship noise using the custom-made software program *Triton* [15]. Blue whale D calls were logged using low-frequency long-term spectral averages and wav files, noting the time period when they occurred, with a resolution of 1 h. Additionally, MFA sonar events, explosions and ship noise were logged with a resolution of 1 minute in the mid-frequency analysis. The detection threshold was set at a signal-to-noise ratio of about 10 dB. The absence of overlap in the frequency ranges for D calls and for the anthropogenic noise sources (between 1–8 kHz) allowed us to have a double-blind experimental design, where low-frequency analysts did not know what mid-frequency analysts logged and vice versa.

### Detection ranges

Source levels of D calls are estimated to be about 160 dB re 1 µPa @ 1 m, even in different populations [20]–[21]. Given that the HARP was deployed at 1300 m depth and the animals are known to call at between 20–50 m depth between foraging dives, animals were recorded above the HARP with ranges up to 8 km radius at the surface. To calculate the blue whale D call detection range, we set a detection threshold of 10 dB above the ambient noise level. Note that MFA sonar levels should not change the probability of D call detection since they do not overlap in frequency. Ship noise, however, is predominantly at low frequencies, and does overlap with the D call in frequency. Thus we would expect that ship noise would decrease the probability of D call detection by masking. Explosions are also low frequency sounds, but because we account for the presence of D calls in one-hour bins, the probability of low-frequency noise masking from explosions during the entire hour is low.

We ran a simulation of the received level at the whale as a function of the distance to the source using a simple model that includes only transmission loss due to spherical spreading and absorption [22] (20 * log_10_ (R)+α * R, where R is the distance in meters and α is the absorption coefficient, −1.9 * 10^−4^ dB/m, for 5 kHz at 15.6°C [23]). For the HARP received levels, we used empirical data from the same basin as the HARP site recorded in the month of July to create a model of sound propagation, accounting for depth. The difference between the received level of MFA sonar and its known source levels [24] indicate that over 97% of the time the sonar sources were farther away than 8 km. Since the recorded foraging blue whales were within 8 km, the uncertainty in the exact positions of the whale and MFA sonar sources is small compared to the long ranges that produced such low MFA sonar received levels. The same holds for explosions, which have high source levels, yet showed moderate received levels at the HARP, suggesting that they were rarely close to the instrument. In contrast, for ships we selected for broadband signals, suggesting that they were relatively close to the HARP and therefore may have a higher received level at the whale than at the HARP, recalling that both the whale and the ship are near the sea surface, whereas the HARP is on the seafloor at 1300 m depth.

### Data analysis

To calculate the probabilities of the animal vocalizing given any anthropogenic noise source (Bayesian statistics), matrices were built with custom code in MATLAB 7 (MathWorks, Natick, MA, US) indicating presence or absence of the animals and MFA sonar, explosions, or ships. The probabilities of the animal vocalizing given the presence of the anthropogenic noise source and the animal vocalizing given the presence of non-anthropogenic noise were calculated, and then the ratio of the first probability divided by the second probability was computed.

To test whether there was a correlation between blue whale D calls and the SPL (in dB re 1 µPa rms) for the frequency band of MFA sonar (1–8 kHz), we first computed the SPL for every second of recording. For every hour we used the maximum SPL obtained and separated the data according to the presence or absence of D calls. Given the binomial nature of our dependent variable (presence or absence of D calls), we ran logistic regressions [16] using the maximum-likelihood method. For this we separated our data into 10 dB SPL classes and calculated the proportion of hours containing D calls for each SPL class. Standard error for each data point was calculated as s.e. = (p * (1−p)/n)^½^, where n is the number of hours analyzed for each data point. We repeated this procedure for the following conditions: MFA sonar, explosions, ship noise, and the remaining hours (labelled as “non-anthropogenic noise” throughout this work). One might think that since our sampling method was continuous, the assumption of independent samples is not fulfilled. In the case of explosions, they consist of short discrete events that usually last well less than one hour. Ship noise typically lasts tens of minutes. MFA sonar events, however, can last for a few hours, but most times the intensity varies between hours. This variation means that consecutive hours will contribute to different sound pressure level bins. Finally, many different non-anthropogenic noise sources can contribute to our “non-anthropogenic noise” situation and may be distributed in any order.

## Supporting Information

Figure S1 **Simulation of received levels and obtained values.** Upper panel shows the sound beam model of the received level at the whale (dashed) and HARP (solid) based on spherical spreading and absorption (whale), and empirical data accounting for depth (HARP). SPL are given in dB re 1 µPa (rms). The sudden drop of 20 dB at about 8 km is due to propagation effects. Lower panel shows a histogram of the SPL (rms) obtained only from the hours containing MFA sonar. Note that the MFA sonar only rarely gets closer than 8 km.
(TIF)
