# Supplementary figures and images for: Blue Whales Respond to Anthropogenic Noise
Source: PLoS One. 2012 Feb 29;7(2):e32681. doi: 10.1371/journal.pone.0032681 (PMC3290562; doi:10.1371/journal.pone.0032681)

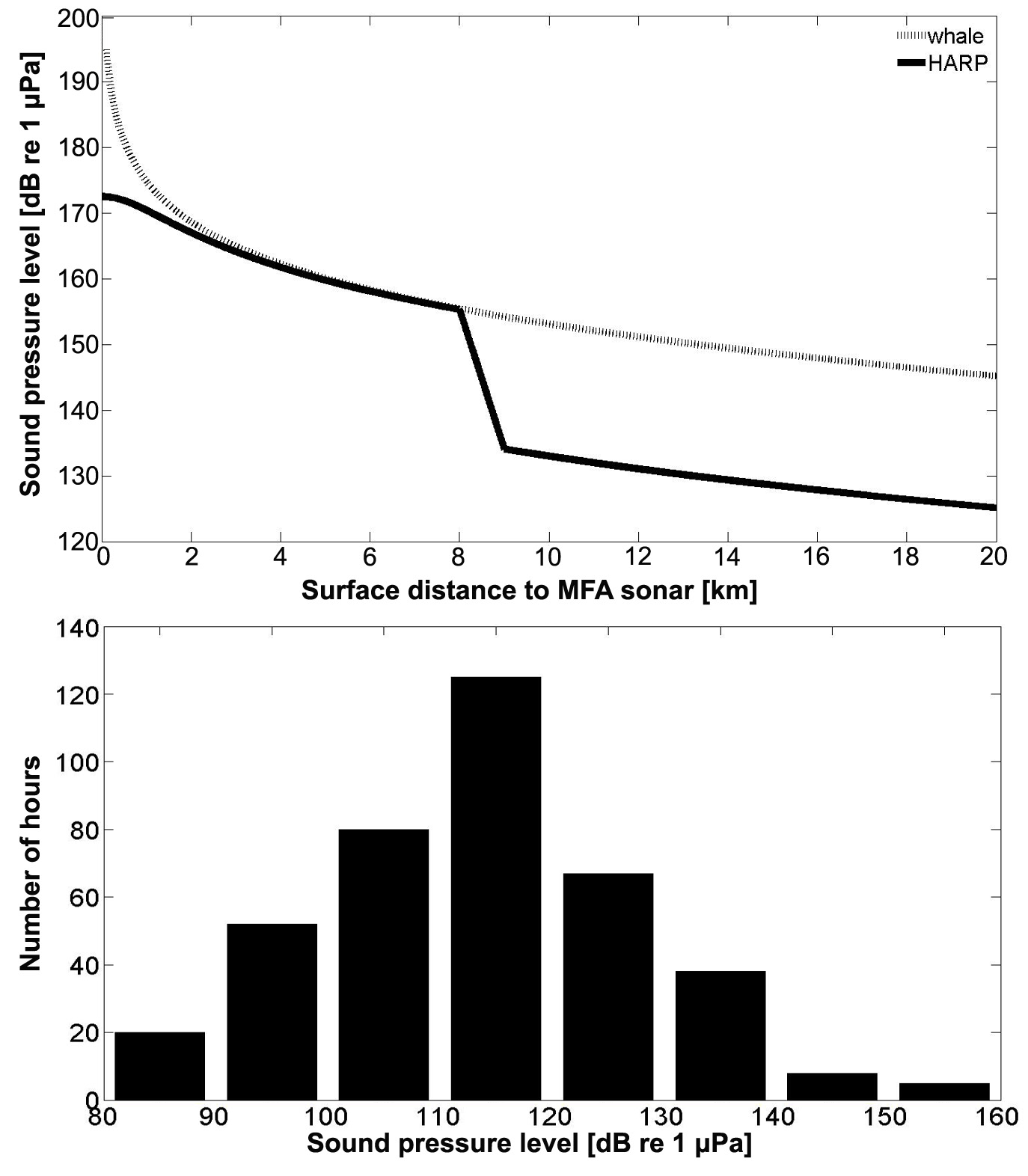

Supplement: Figure S1 — Simulation of received levels and obtained values. Upper panel shows the sound beam model of the received level at the whale (dashed) and HARP (solid) based on spherical spreading and absorption (whale), and empirical data accounting for depth (HARP). SPL are given in dB re 1 µPa (rms). The sudden drop of 20 dB at about 8 km is due to propagation effects. Lower panel shows a histogram of the SPL (rms) obtained only from the hours containing MFA sonar. Note that the MFA sonar only rarely gets closer than 8 km. (TIF) [file pone.0032681.s001.tif]
